# Individuals’ preferences for future biological sample and genomic data sharing in the Australian Reproductive Genetic Carrier Screening Project

**DOI:** 10.1038/s41431-026-02048-3

**Published:** 2026-02-25

**Authors:** Matilda A. Haas, Evanthia O. Madelli, Martin B. Delatycki, Edwin P. Kirk, Tiffany F. Boughtwood

**Affiliations:** 1 Australian Genomics, Parkville, VIC Australia; 2 Murdoch Children’s Research Institute, Parkville, VIC Australia; 3 Victorian Clinical Genetics Services, Parkville, VIC Australia; 4 Sydney Children’s Hospital, Randwick, NSW Australia; 5 NSW Health Pathology, Randwick, NSW Australia

**Keywords:** Genetics research, Ethics

## Abstract

Genomic information collected in research settings is a valuable resource that can be shared for future (secondary) research with the consent of the individual. Whether individuals participating in genomic research are comfortable with broad consent and all research sharing scenarios is largely unknown. The Australian Reproductive Genetic Carrier Screening Project (Mackenzie’s Mission) investigated the feasibility and acceptability of population reproductive carrier screening for severe recessive genetic conditions occurring in childhood. Enrolment and consent for participation was completed digitally using an online Portal or REDCap. Consent included an option to complete ten specific questions about preferences for future research use of samples and data. Preferences for future research were completed by 23.5% (4288) of individuals. The remaining 76.5% gave broad consent to data sharing. Those who chose to complete the questions shared similar demographics to the rest of the cohort. Individuals were most permissive of sharing with not-for-profit (78.0%) and university (78.2%) research organisations, for general (79.8%) and health / medical / biomedical research (82.2%). People were less likely to consent for use by governments (59.2%) and commercial organisations (33.7%). Nearly 60% of people want to be notified every time their data is shared. Updates to consent preferences were made 1785 times, by 282 people. This study supports the need for research programmes to facilitate flexible models of consent, including specific and dynamic consent. It also demonstrates a scalable model in which participant-led choices contribute to reduced ambiguity about data sharing permissions.

## Introduction

Genomic data sharing is invaluable in progressing our understanding of human genetic variation and its contribution to health and disease [1]. However, ethical frameworks require consent of the individual for sharing re-identifiable genomic information collected in research settings for future (secondary) research. Genomic research has typically relied on broad consent for non-specified future data sharing, asking participants to provide consent for sharing for research approved by a Human Research Ethics Committee (or jurisdictional equivalent).

Whether individuals participating in genomic research are comfortable with all research sharing scenarios is largely unknown. For example, research participants may vary in their willingness for their data to be transferred outside their own country, used by organisations for research with commercial intentions, or accessed by government entities for research purposes. Preferences are likely to depend on social, cultural, personal, and other factors [2]. Research about people’s preferences for genomic data sharing has focussed on obtaining public views [3–6] or have been hypothetical studies or surveys of patient or research populations [7–9]. Studies that capture a research population’s actual choices in projects they are participating in may be more relevant to understanding preferences for data sharing [10, 11]. Individuals and carers experiencing a rare disease have been reported to be more willing to share data, often motivated by the potential for discovery of a cure [7], as are those with a history of cancer [8], while those without a history of disease are relatively unwilling to share their genomic data [4].

We previously published research about consent preferences for biological sample and data sharing in a cardiovascular genetic disease research cohort ( < 100 people) and found that the research participants were generally altruistic when it came to data sharing [12]. More than 80% were willing to share their data for general research use, most favourably to universities and not-profit organisations The delivery of dynamic choices about future research sharing was based on Data Use Ontology, a product of the Global Alliance for Genomics and Health (GA4GH) [13] and enabled by using CTRL (control), an online dynamic consent tool [14]. Real-world genomic research studies have rarely asked participants for their consent preferences for future use of their data in such detail.

Here, we have expanded our research to a population cohort – the Australian Reproductive Genetic Carrier Screening Project (Mackenzie’s Mission). Mackenzie’s Mission investigated the feasibility and acceptability of a national reproductive genetic carrier screening programme. The programme gave couples information about their chance of having a child with a severe and/or life-limiting genetic condition occurring in childhood [15]. CTRL was adapted and extended to provide an education and consent portal for couples to participate in Mackenzie’s Mission. As part of this, they could choose to give preferences for future access to samples and data use. This study reports on these preferences.

## Materials and methods

The Mackenzie’s Mission study, including its inclusion criteria and outcomes have been previously described [15, 16]. Couples were invited to participate in Mackenzie’s Mission by their healthcare provider. The CTRL platform was extended upon to develop the Mackenzie’s Mission Participant Portal (‘the Portal’) for individuals to enrol in the study and provide their consent. The data was automatically transferred to a REDCap database [17, 18]. Depending on the couples’ needs, alternative pathways were developed in REDCap to assist them. During a pilot phase, couples enroled directly via REDCap with the assistance of the study team. As part of the enrolment process, each member of the couple was required to individually review the study information and education materials and complete the consent form.

### Study population

Briefly, 9255 couples completed enrolment in the study, recruited by 775 healthcare professionals across all states and territories in Australia within a period of 2.5 years (November 2019–March 2022). Of the 9255 enrolled couples, 9107 couples accepted genetic carrier screening and were provided with a result. Of those, 9106 couples (18,212 individuals) were included in the initial analysis for this study.

### Defining cohorts by enrolment method

Individuals and their consent preferences were initially separated into four cohorts by the enrolment method: (1) ‘Portal with assistance’, (2) ‘Portal no assistance’, (3) ‘REDCap no assistance’, and (4) ‘REDCap pilot’.

### Preferences for return of results and future sample and data use

An optional section called ‘Review consent preferences for research outside this study’ was developed in the Portal and the REDCap enrolment database. Participants had the option to edit their responses in this section at any time and as many times as they wanted to during the study period. Couples that enrolled via an alternative pathway, as outlined above, completed this section directly in REDCap.

Participants could select optional responses (‘Yes’, ‘No’, ‘Unsure’) to 10 consent questions about their preferences for future research use. Data analysis included: completion rate; number of users that accessed the section and exited without making changes; preferences selected; whether preferences were updated (including number of times); what the updated preferences were; timing of updates since initial enrolment; whether changes in preferences coincided with specific events or study milestones; participant enquiries about sample and data management and sharing, and what choices people who made these enquiries ultimately made about future sharing.

### Data analysis

Data cleaning processes included a quality check to ensure only couples who completed screening were included. Descriptive statistics were used to analyse all participant preferences and demographics. Pearson’s chi-square tests were used to determine the relationship between result disclosure and change in preferences as well as differences in demographics. Chi-square goodness for fit tests were performed in StataC 17.0 [19].

To extract participant enquiries relevant to this study, the enquiries log was searched for terms: ‘Data’ ‘Share’ ‘Sharing’ ‘Release’ ‘Releasing’ ‘Genetic information’ ‘Future studies’ ‘Future research’ ‘Future project’s’ ‘Other studies’ ‘Other research’ ‘Other projects’ ‘Privacy’ ‘Storage’. Participant enquiries were analysed qualitatively. Two reviewers (MH, EM) separately coded and reached consensus on codes that were then used to develop themes.

## Results

### Cohort demographics

**Fig. 1:**
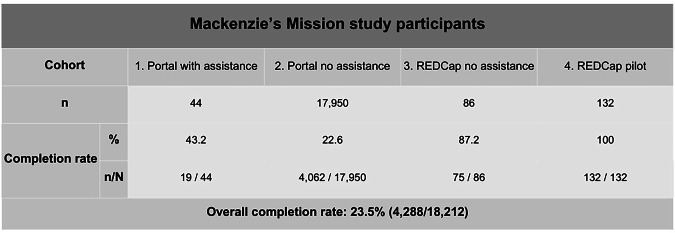
Cohorts and completion rates for future sample and data sharing page. ‘REDCap no assistance’ completion rates may have been higher because the question page was part of the recruitment flow rather than a page optionally entered from a dashboard. n number.

Detailed demographic information of the overall Mackenzie’s Mission cohort has been reported [16]. Individuals who optionally completed their preferences for future use of samples and data accounted for 23.5% (4,288) of the overall cohort (18,212) (Fig. 1). The remaining 76.5% of participants who did not complete the granular preferences questions instead provided broad consent for future use of data on a different page in the consent portal. Most participants were in the age range 30–34 (1,555). The cohort consisted of 45.6% of males (1954) and 54.4% of female (2,334) participants. The most frequent education level was a Bachelor degree (1,734) and the most frequent represented ancestry was European. The proportion of individuals who were Aboriginal or Torres Strait Islander was 1.5% (47), compared with 1.1% of the overall Mackenzie’s Mission cohort (not significant, Kirk et al., 2024). Most participants spoke English only, comprising 84.7% (3,630) of the cohort, while fewer spoke English and other languages (10.1%, 432) and languages other than English only (5.3%, 226) (Table 1).

**Table 1:** Demographics for the 4,288 individuals included in this study.

Characteristic	%(n)
**Age**	
Under 25	0.84 (36)
25–29	11.1 (475)
30–34	36.3 (1,555)
35–39	35.4 (1,519)
40 or over	16.4 (703)
**Genetic sex**	
Male	45.6 (1,954)
Female	54.4 (2,334)
**Education**	
Bachelor degree	40.4 (1,734)
Certificate	19.6 (841)
Did not complete Year 12	3.6 (154)
Other	4.4 (187)
Post-graduate	24.4 (1,045)
Year 12	7.6 (327)
**Ancestry**	
Europe	71.8 (3,079)
Oceania	7.8 (333)
Asia	15.1 (649)
Middle East	3.9 (167)
Africa	2.1 (88)
North America	0.4 (15)
Central or South America	1.9 (82)
**Aboriginal or Torres Strait Islander**	
Yes	1.5 (47)
No	98.5 (3,116)
**Language(s)**	
English only	84.7 (3,630)
English and other language(s)	10.1 (432)
Language(s) other than English	5.3 (226)

n number.

### Establishing sub-cohorts

The cohort who optionally completed their preferences for future use of samples and data was derived from four separate cohorts due to differences in recruitment processes (Fig. 1). Cohorts included: (1) ‘Portal with assistance’: 44 individuals who completed the self-directed registration and consent using the Portal but contacted research personnel (genetic counsellor or general enquiries support) with sample and/or data related enquiries, (2) ‘Portal no assistance’: 17,950 individuals who completed the self-directed registration and consent using the Portal without seeking any assistance from research personnel, (3) ‘REDCap no assistance’: 86 individuals who were provided with the REDCap version of the Portal, usually because they experienced a technical issue with the Portal, and (4) ‘REDCap pilot’: 132 individuals who sat together with research personnel while working their way through the REDCap version of the Portal.

### Completion rates—future use of data

Completion was defined as making one or more optional selections on the future use of data page. Individuals using the Portal optionally accessed this page via the dashboard. For the ‘Portal with assistance’ group, choices were made by 43.2% (19/44) of individuals and for ‘Portal no assistance’ choices were made by 22.6% (4,062/17,950) (Fig. 1). For individuals using the Portal (either in the with assistance or no assistance groups) 77.3% (13,913/17,994) did not make selections for future use of data. Of those, 91.3% (12,697/13,913) participants did not enter the page and therefore did not review the questions, while 8.7% (1,216/13,913) entered the future use of samples and data page but left without making any selections, which automatically saved their answers as ‘Unsure’. These answers were excluded from further analysis. Individuals in these participant groups instead provided broad consent to future data sharing on a different page in the consent portal.

Participants who enrolled via REDCap were guided through enrolment pages step by step, meaning that all participants landed on the consent for future research use page. For the ‘REDCap no assistance’ group, the completion rate was 87.2% (75/86). Individuals in the ‘REDCap pilot’ group, who also had study personnel guiding them through consent, made selections for these questions at a 100% completion rate (132/132) (Fig. 1). The completion rates for Partner 1 and Partner 2 and their consent choices are shown in Supplementary Tables 1, 2.

### Preference for future sample and data use

**Fig. 2:**
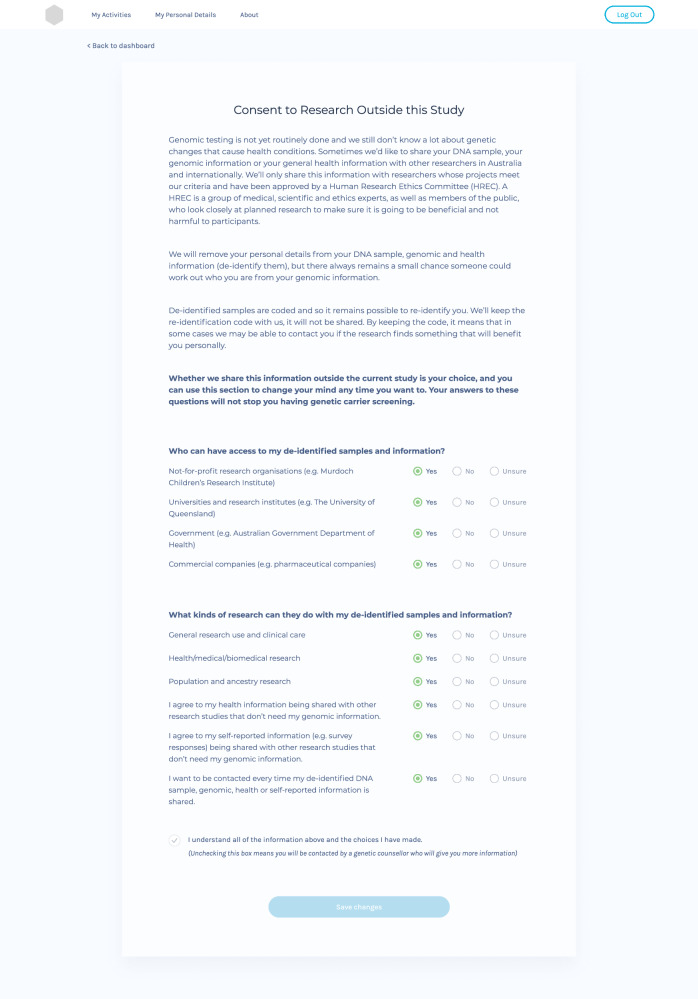
Participant view of the sample and data sharing page in the Mackenzie’s Mission Portal.

**Fig. 3:**
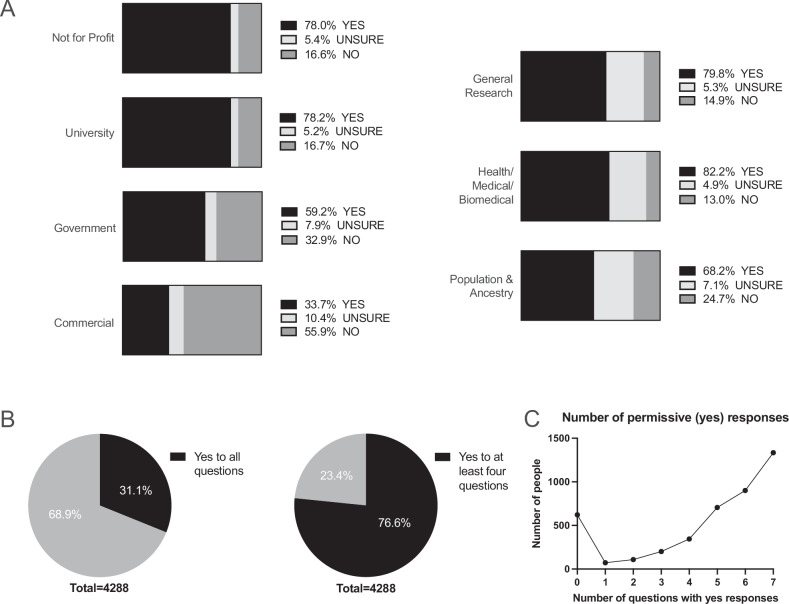
Final preferences for biological sample and data sharing. **A** Final selections for permitted organisations (left) and research uses (right). All four cohorts are combined. **B** The percentage of individuals who provided a ‘Yes’ response to all questions (left) and the percentage of individuals who recorded mostly permissive responses (‘Yes’ to at least four of seven questions). **C** The number of individuals who provided a ‘Yes’ response to each number of questions.

Individuals provided answers to questions about permission for future use of their biological samples and data (Fig. 2, Haas et al. 2021; 2024). Combining all four cohort groups, the final preferences of individuals were: 78.0% (3,344/4,286) permitted use of their samples and data by not-for-profit organisations and 78.2% (3,350/4,286) permitted use by universities, while 59.2% (2,538/4,288) gave permission for use by governments and 33.7% (1,444/4,286) to the commercial sector (Fig. 3A and Supplementary Tables 3, 4).

When asked about types of research, 79.8% (3,420/4,287) of individuals were willing to share for general research use and clinical care and 82.2% (3,523/4,287) for health/medical and biomedical research, with 68.2% (2,922/4,285) agreeing to share for population and ancestry research (Fig. 3A and Supplementary Table 3).

Patterns of permissive responses showed that 31.1% (1,334/4,288) individuals provided a ‘Yes’ response to all questions (Figs. 3B), and 76.6% (3,285/4,288) provided mostly permissive responses (a ‘Yes’ response to at least four out of seven questions). The number of people who provided ‘Yes’ responses to zero through to seven questions is shown (Fig. 3C). For organisations permitted access, participants were least likely to provide a ‘Yes’ response to commercial use of data. Many participants who recorded a ‘Yes’ response to commercial use also agreed to all other organisations (32.5%; 1,391/4,286) (Supplementary Fig. 1).

Overall, 57.5% (2,464/4,287) of people want to be notified every time their information is shared, 60.1% (2,576/4,287) agreed to health information being shared with studies that do not need their genomic information, and 61.5% (2,635/4,287) agreed to their self-reported information (such as survey responses) being shared for research without their genomic information (Supplementary Table 3). The comparison between this population screening study and the cardiovascular rare disease cohort previously published is shown in Supplementary Table 5.

The ‘REDCap pilot’ group was guided through the consent process by study personnel. Although this meant they had more opportunity to ask questions about sample and data sharing, this did not appear to influence the number of ‘Yes’ responses, which was 69.3% (914/1,319) compared to 65.8% (28,216/42,866) of ‘Yes’ responses made by all groups combined in ‘overall’. However, the percentage of ‘Unsure’ responses was lower than that of any other group (2.5%, compared with 7.2% ‘Portal no assistance’, 5.6% ‘REDCap no assistance’, and 12.1% ‘Portal with assistance’, Supplementary Table 3).

### Updates to consent preferences

Individuals were able to log back into the Portal and make changes to their choices at any time during the study period. From the two Portal groups who made initial selections in the future sample and data sharing page, 6.7% (272/4,081) individuals made a total of 1766 changes to choices during the study period with a median of 7 (range 1–29) changes per individual. The most frequent change was from ‘Unsure’ to ‘Yes’, and the least frequent change was from ‘No’ to ‘Unsure’ (Table 2).

**Table 2:** Updated responses by question.

Data use permission	Total number of changes to response	No to Unsure	No to Yes	Unsure to No	Unsure to Yes	Yes to No	Yes to Unsure
**Not-for-profit**	176	1	7	33	**111**	16	8
**University**	180	2	9	37	**105**	20	7
**Government**	187	4	9	62	**88**	16	8
**Commercial**	182	8	14	**98**	44	13	5
**General Research**	180	1	4	26	**114**	25	10
**Health, Medical, Biomedical research**	179	2	3	24	**117**	23	10
**Population ancestry**	170	3	5	42	**92**	19	9
**Health data**	173	1	5	54	**91**	13	9
**Self-reported data**	176	1	7	44	**103**	12	9
**Notify individual**	182	1	18	64	**80**	11	8
**Total**	1785	24	81	484	**945**	168	83

The four cohorts are combined. The cell with the most frequent change for each question is highlighted with bold text.

**Fig. 4:**
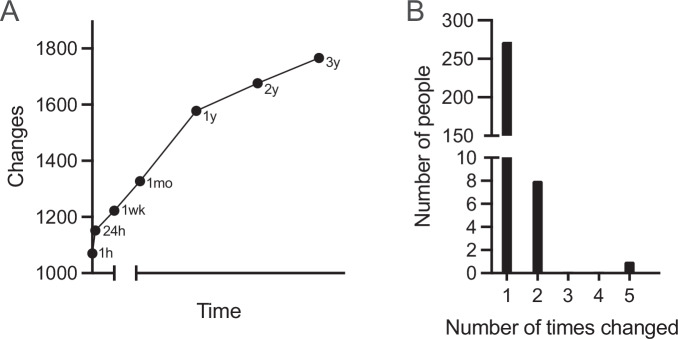
Changes to preferences. **A** The cumulative number of changes to choices over time. Most changes to choices were made within a timeframe consistent of their first session. **B** The number of individuals who made changes to a single question. Most participants changed their choice once.

There were 1070 changes made within the first hour of making the first selection, indicating they were likely made in the initial session in the Portal. This is 60.6% of all changes. Cumulatively, the changes were made within 24 h (1,152), a week (1,222), 1 month (1,327), 1 year (1,578), 2 years (1,676) and 3 years (1,766) from the initial responses (Fig. 4A). Eight individuals made changes twice, and one individual made changes five times (Fig. 4B).

Receiving the test result, either ‘increased chance’ or ‘low chance’ of having a child with a severe recessive genetic condition, appeared to trigger changes to choices about future research use of samples and data. Of the 1,785 total changes made by Portal and REDCap groups combined, 202 changes were made by 42 individuals within 7 days after the date of the result disclosure (*p* = 0), indicating that a statistically significant proportion of individuals changed their choices after result disclosure. Ninety-two days prior to the shutdown of the Portal at the end of the study, all participants received email notification indicating that their preferences as of the shutdown date were final. During the time between the email and the shutdown, a total of 219 changes were made by 43 individuals (*p* = 0), indicating that a statistically significant proportion of individuals changed their choices after receiving the alert that the study was closing.

There did not appear to be any activity following a high-profile commercial telecommunications breach involving personal data after news broke on the 22nd of September 2022 [20]. One person made changes to choices in the week following news breaking of an insurance provider’s health data breach on 13th October 2022 [21]. The individual changed five answers, all becoming more restrictive of sample and data sharing.

### Participant enquiries about data and future sample and data use preferences

The ‘Portal with assistance’ group was comprised of 22 couples (i.e., 44 individuals) where at least one partner in the couple had made an enquiry to the study team related to data sharing, either to the genetic counsellor or via email/phone. Twenty-one enquiries were coded. Most frequently, enquiries related to data storage and use, the data retention period, data sharing, data privacy, and storage of the DNA sample (Table 3).

**Table 3:** Enquiries about data and future research. Data and future research related enquiries made by 22 couples were coded and categorised.

Code	Number of enquiries
**Data**	
Data storage	8
Data use	8
Data retention period	6
Data sharing (including third parties and selling data)	6
Data privacy	5
Data sharing (legal)	3
Data destruction	3
Data security	2
Data de-identification	2
Re-identification concerns	1
**DNA Sample**	
Sample storage	5
Sample destruction	1
Sample retention period	1
**Consent**	
Consent form update without re-contact	1
Consent withdrawal	1
Data use for promotional material without consent	1
**Other**	
Insurance	4
Dissemination of results	1
Notification of future data use	1
Service provider trust	1

Nineteen of 44 individuals in this group made choices in the future sample and data sharing section. This cohort made more restrictive data sharing decisions for every question than the overall cohorts combined: total ‘Yes’ selections were 26.3% (50/190), compared to 65.8% (28,216/42,866) in all cohorts combined (*p *= 0) and ‘No’ selections were 61.6% (117/190), compared to 27.1% (11,623/42,866) in all cohorts combined (*p* = 0). The percentage of individuals who wanted to be informed every time their data was shared was 78.9% (15/19) for the ‘Portal with assistance cohort’, compared with 57.5% (2,464/4,287) for all cohorts combined (*p* = 0.059; Supplementary Tables 3, 4).

## Discussion

This research explored the preferences of individuals participating in a population genomics study about future use of biological samples and data. In summary, optional questions about preferences for future use of samples and data were completed by 23.5% of individuals in the overall Mackenzie’s Mission cohort. They were more permissive to sharing with not-for-profits and universities than government or commercial organisations, for general and health / medical / biomedical research purposes. Permission rates were similar to a previous cardiovascular genetic disorders cohort [12]. Updates to preferences were mostly made within the time frame of the initial consent session (60.6%), but some changes were made up to three years later. A significant number of people made changes in the seven days following test result disclosure or notification of shutdown of the study Portal. Individuals who were part of a couple that made an enquiry to the study related to data / future use made more restrictive decisions for sharing samples and data.

Our study was able to test preferences for specific versus broad consent for future use of samples and data. Just under one quarter (23.5%) of participants made specific granular selections about future research, while the other three quarters did not, despite the option being available to them. This may be indicative of a smaller proportion of people wanting to have more specific control over sample and data sharing. However, we observed completion rates of optional future research consent were higher in REDCap cohorts (e.g. 87.2% in the REDCap no assistance group). This was almost certainly because consenting individuals saw the questions in REDCap as part of the enrolment flow (even though the page description clearly indicated that completion was optional). This contrasted with the Portal cohort who had to enter the page from the dashboard item that was marked optional and located below the ‘view result’ section rather than with the other enrolment sections on the dashboard. Therefore, simply making the questions visible to people as part of the consent flow was associated with a higher completion rate. This may indicate that broad consent was not necessarily an active preference of participants in the study and should be taken into account when designing online consent strategies.

There is growing literature about participant views on preferences for broad versus more specific consent models. A recent systematic review on patient preferences for sharing of personal health information and digital consent found that 71% of participants prefer granular, informative, and transparent consent choices [22]. An older systematic review reported that broad consent was supported by Americans—but only when it was the only option offered to them. The review also found that characteristics associated with being favourable to broad consent included being male, white, older and more affluent [23]. In Japan, a public survey found 60.9% of people preferred specific or dynamic consent for biobank research [24]. In contrast, there are examples in the medical ethics literature where the conclusion has been that broad consent is a better model than study specific consent, for example for biobanks [25]. It will be important to consider the specific research and consent contexts and participant population when designing consent. Ultimately, flexible options, such as offered in our study, will accommodate the needs of individuals.

Our research supports that different research cohorts and populations will value the opportunity for more specific and dynamic consent differently. Although not directly comparable due to different study designs, 23.5% of this cohort chose to make dynamic consent choices, in contrast to 15% of a previously reported rare disease cohort [12]. It has been demonstrated that the rare disease community are highly motivated to participate in research opportunities presented to them [26] and altruistic about sharing their data for research purposes [27], even if it will not necessarily benefit them directly [28]. However, when we compared the healthy Mackenzie’s Mission population and rare disease cohorts, we found that the population cohort were also permissive toward the same research organisations and types of research. The more restrictive choices observed in response to some questions, like commercial use, were more pronounced in the population cohort. This can be considered alongside a large international sample of the public with little experience of genetics, who indicated more restrictive views about genomic data sharing than either of our research cohorts [4]. Our data support observations that familiarity with health research and/or genetics is associated with more permissive views about genomic data sharing. While people are willing to share their data, there is increasing evidence that research and biobank participants want to know what their data is being used for [12, 29], which is important for trust [30].

One hesitancy with the implementation of dynamic consent models is that if most people choose not to give permission for data sharing, data sharing efforts may be limited. Our research shows that this is not the case since around 80% of participants agree to share data, and this choice is stable over time [12, 31]. In the CHRIS Study, one of the largest ongoing studies using dynamic consent, 22 participants (of around 13,000 enrolled) changed their choices between 2011 and 2018 and changes were made a median of 18 days after initial consent [32]. However, there are times along the research journey that participants review their consent decisions, especially if prompted. Our study also spans a reasonably short time frame (approximately 3 years), so it is possible that the circumstances of an individual would not change very much during that time. The value of dynamic consent may be increased for research that spans decades.

Individuals who were part of a couple who made enquiries about data and future use of data made more restrictive choices. For example, 82% of the whole cohort agreed to sharing for health, medical and biomedical research, while just 32% of those who made data related enquiries agreed. There is no indication that participants in the study were unsatisfied with the responses by study personnel. Rather, we conclude that those who had data sharing concerns were likely to make restrictive choices anyway. Importantly, these concerns did not stop them from participating altogether. This ‘Portal with assistance’ cohort had the highest percentage of ‘Unsure’ answers at the end of the study. Those in the ‘REDCap pilot’ cohort, who had study personnel with them during enrolment, did not make more permissive or restrictive choices than cohorts who made their choices independently, which implies that recruitment practices in this study were not influential on future research choices. However, unassisted cohorts did have more ‘Unsure’ answers than the ‘REDCap pilot’ cohort.

Another concern about digital and dynamic consent has been that it is only available to those who have access to technology and the internet, and are technology literate, and this could exacerbate the ‘digital divide’ [33, 34]. This study did not address this directly. People making selections about future use of samples and information were most often Bachelor educated, which was consistent with the overall cohort, but higher than the national rate (40% for this study compared with 32% of Australians aged 15–74) [35]. There was a slightly higher but not significant percentage of Aboriginal and Torres Strait Islander participants that provided consent using the future data sharing questions than were represented in the overall cohort. This finding may support further exploration of the acceptability and desirability of this approach to consent, with a focus on engaging with Aboriginal and Torres Strait Islander peoples to design appropriate ways to provide consent for health and genomic data sharing.

### Limitations

One limitation was that the overall high technology literacy of the individuals involved meant that we could not explore the barrier of technological literacy. We had previously used analytics to understand the use characteristics of digital consent tools, for example number of sessions, session length and page views [12], but analytics were not able to be linked for this study. Also, to understand people’s preferences and views about providing specific consent for future research use of samples and data, pairing the data analysis presented here with qualitative research through interviews would be informative. One area of further research may include whether participants understood what was meant by sharing of their genetic data for future research, their level of awareness that this could involve sharing their individual genetic data rather than their combined couple results, and whether more explicit knowledge would impact their data sharing choices.

### Future directions

Future use of samples and data questions based on DUO have been applied to two applications of CTRL [12] (and the current study). While we can infer that individuals understood and were able to make consent selections, a refined version should be applied in future. A newer version would also benefit from the use of plain language and examples of data use. For example, it is possible that individuals may be more permissive of government and commercial access if they knew more about what kinds of research these organisations may be doing. Another objective will be to integrate CTRL with data access and release platforms to further facilitate data transfers data sharing according to the real-time preferences of individuals.

## Conclusions

Due to the cohort size, this study adds a great deal to our understanding of individual preferences for use of samples and data by people participating population genomic research, and the use of digital tools to make those decisions. It shows the need for flexible options for consent and specific and dynamic consent to be made available to those who want it. This study provides information that could contribute to planning of future genomic research studies and their consent and data governance frameworks. It also shows that data sharing ontology can be implemented as participant-led choices in health genomics research, which reduces ambiguity about data sharing permissions.

## Supplementary information

Supplementary information

## Data Availability

Data from this study is available via contacting Australian Genomics to submit a data access request.

## Code availability

The CTRL code, which provided the basis for the Mackenzie’s Mission Portal, is publicly available at https://github.com/garvan-Data-Science-Platform/CTRL.
