# Supplementary material for: Individuals’ preferences for future biological sample and genomic data sharing in the Australian Reproductive Genetic Carrier Screening Project
Source: Eur J Hum Genet. 2026 Feb 25;34(7):938–46. doi: 10.1038/s41431-026-02048-3 (PMC13342307; doi:10.1038/s41431-026-02048-3)
Supplement: Supplementary file 1 — Supplementary information [file 41431_2026_2048_MOESM1_ESM.docx]

|  | Partner 1 %(n) | Partner 2 %(n) | Overall %(n) |
| --- | --- | --- | --- |
| 1. Portal (with assistance) | 41 (9) | 45.5 (10) | 43.2 (19) |
| 2. Portal (no assistance) | 24.7 (2,221) | 20.5 (1,841) | 22.6 (4,062) |
| 3. REDCap (no assistance) | 82.9 (34) | 91.1 (41) | 87.2 (75) |
| 4. REDCap pilot | 100 (66) | 100 (66) | 100 (132) |
| Overall | 25.6 (2,330) | 21.4 (1,958) |  |

Supplementary table 1. Completion rates by partner.

Supplementary table 2. Choices by Partner. Overall percentage of ‘Yes’ responses is shown.

Supplementary table 3. Final preferences for future research. Number of responses to questions about future research use of samples and data, by cohort. Overall number of responses by all cohorts combined and percentages.

Supplementary table 4. Responses to individual questions by cohort (%)

| Data use permission | Population cohort  Yes responses (%) | Rare disease cohort*  Yes responses (%) |
| --- | --- | --- |
| Not-for-profit | 78.0 | 80.2 |
| University | 78.2 | 82.4 |
| Government | 59.2 | 70.3 |
| Commercial | 33.7 | 45.1 |
| General Research | 79.8 | 80.2 |
| Health, Medical, Biomedical research | 82.2 | 82.4 |
| Population ancestry | 68.2 | 68.1 |
| Health data | 60.1 | 59.3 |
| Self-reported data | 61.5 | 58.2 |
| Restricted to my condition | NA | 44.0 |
| Notify individual | 57.5 | 63.7 |

Supplementary table 5. Comparison between the use permissions of the Mackenzie’s Mission cohort and a cardiovascular genetic disorders cohort (*Haas et al., 2024). NA = not applicable.


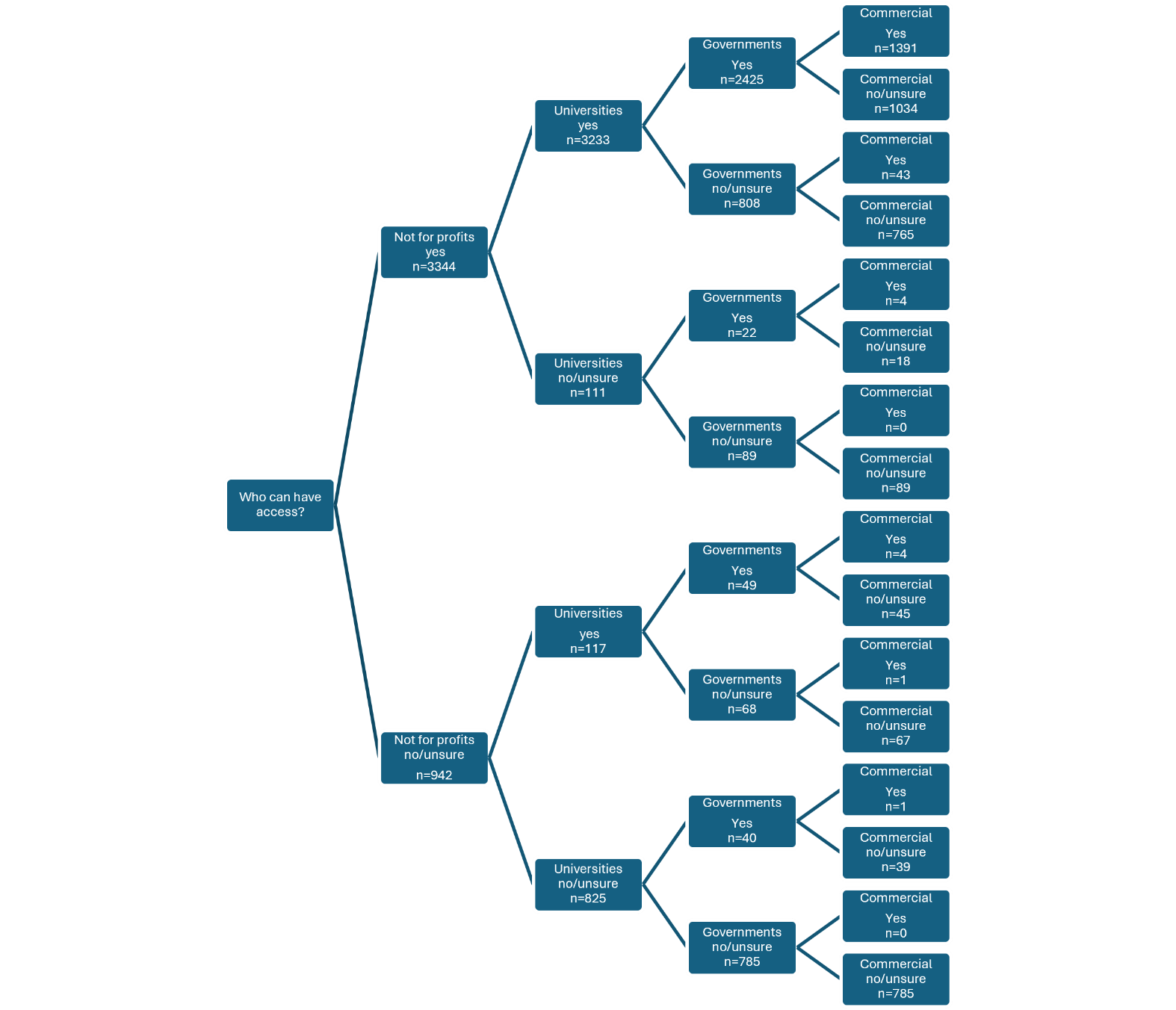


Supplementary figure 1. Mapping response to the question ‘who can have access to my de-identified samples and information?’
